# An energy-efficient pathway to turbulent drag reduction

**DOI:** 10.1038/s41467-021-26128-8

**Published:** 2021-10-04

**Authors:** Ivan Marusic, Dileep Chandran, Amirreza Rouhi, Matt K. Fu, David Wine, Brian Holloway, Daniel Chung, Alexander J. Smits

**Affiliations:** 1 Dept. of Mechanical Engineering, University of Melbourne, Melbourne, VIC Australia; 2 Dept. of Engineering, School of Science and Technology, Nottingham Trent University, Nottingham, UK; 3 Graduate Aerospace Laboratories (GALCIT), Caltech, Pasadena, CA USA; 4 Intellectual Ventures, Bellevue, WA USA; 5 Dept. of Mechanical and Aerospace Engineering, Princeton University, Princeton, NJ USA

**Keywords:** Aerospace engineering, Mechanical engineering

## Abstract

Simulations and experiments at low Reynolds numbers have suggested that skin-friction drag generated by turbulent fluid flow over a surface can be decreased by oscillatory motion in the surface, with the amount of drag reduction predicted to decline with increasing Reynolds number. Here, we report direct measurements of substantial drag reduction achieved by using spanwise surface oscillations at high friction Reynolds numbers (${{{\mathrm{Re}}}_{\tau }}$) up to 12,800. The drag reduction occurs via two distinct physical pathways. The first pathway, as studied previously, involves actuating the surface at frequencies comparable to those of the small-scale eddies that dominate turbulence near the surface. We show that this strategy leads to drag reduction levels up to 25% at ${{{{{{{{\mathrm{Re}}}}}}}}}_{\tau }$ = 6,000, but with a power cost that exceeds any drag-reduction savings. The second pathway is new, and it involves actuation at frequencies comparable to those of the large-scale eddies farther from the surface. This alternate pathway produces drag reduction of 13% at ${{{{{{{{\mathrm{Re}}}}}}}}}_{\tau }$ = 12,800. It requires significantly less power and the drag reduction grows with Reynolds number, thereby opening up potential new avenues for reducing fuel consumption by transport vehicles and increasing power generation by wind turbines.

## Abstract (web summary)

The speed and efficiency of transportation and energy systems, including airplanes, ships, and wind turbines can be limited by skin-friction drag. The authors describe a pathway to drag reduction by controlling the large-scale turbulent eddies occurring away from the surface for improved function.

## Introduction

Despite significant progress in uncovering the origins of turbulence^1–6^, many longstanding scientific and engineering difficulties remain in controlling turbulence under conditions relevant to critical transportation and energy applications. In flows encountered by airplanes, ships, wind turbines, and pipelines, for example, the skin-friction drag generated by turbulence constrains both speed and fuel efficiency. Even modest reductions in drag could yield significant economic and environmental benefits, such as improvements to the fuel efficiency of large vehicles and the power capacity of wind turbines.

**Fig. 1:**
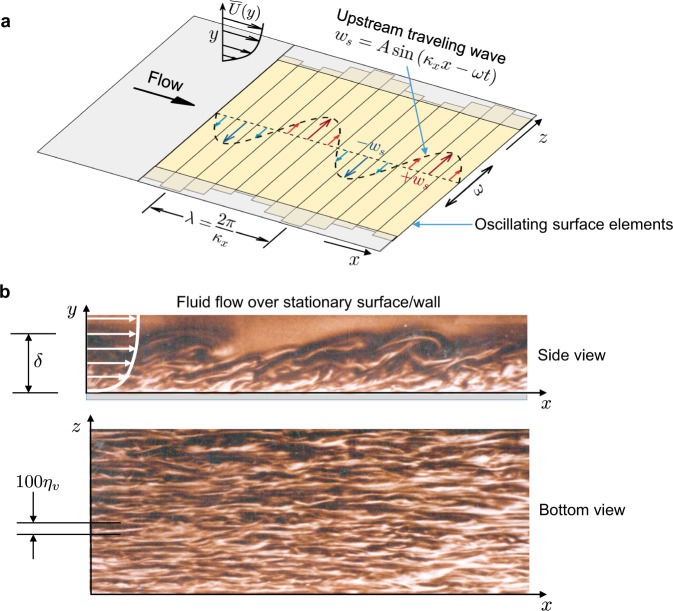
Oscillating surface and turbulent flow scales. **a** Schematic of the actuation scheme: an upstream travelling wave of spanwise velocity *w*_*s*_ and wavelength *λ* is created by synchronously sliding elements (slats) embedded in the surface at a frequency *ω*, interacting with a boundary layer flow with mean velocity profile $\overline{U}(y)$. **b** Distinct regions of large- and small-scale turbulence are evident in this visualization of fluid flow at ${{{{{{{{\mathrm{Re}}}}}}}}}_{\tau }=500$ over a stationary surface (courtesy of D. Coles). (Top) A side view reveals how, in outer regions (farther from the surface), large-scale motions scale with the thickness *δ* of the boundary layer. (Bottom) In the corresponding view through the boundary layer from below, small-scale streamwise streaks are prominent close to the surface and have an average spanwise spacing of 100*η*_*v*_, where ${\eta }_{v}=\nu /{{u}_{\tau }}_{0}$ is the viscous length (ref. ^27^; see text).

One of the most promising candidates for significantly reducing drag is spanwise oscillation of surface elements synchronized to produce a travelling wave in the direction opposite to that of the fluid stream, as shown in Fig. 1a^7–18^. The sinusoidal oscillation is prescribed by

1 $${w}_{s}(x,t)=A\sin ({\kappa }_{x}x-\omega t),$$

in which *w*_*s*_ is the instantaneous (spanwise) wall velocity, *A* is its amplitude, *ω* is the angular frequency of spanwise oscillations, and *κ*_*x*_ = 2*π*/*λ* is the streamwise wavenumber of the travelling wave. Here *x*, *y*, and *z* denote the streamwise, wall-normal, and spanwise coordinates, respectively, and *t* is time. The oscillating elements act on the turbulent fluid flow, which displays intense streaky motions near the surface and larger, billowy motions away from the surface (Fig. 1b). Numerical simulations of this kind of active-surface control have demonstrated impressive levels of drag reduction (DR) up to 50%^13^. DR is measured during active flow control as the fractional decrease in $\overline{{\tau }_{w}}$, the time-averaged local drag force per unit area acting on the surface or "wall”, given by

2 $${{{{{{{\rm{DR}}}}}}}}=\left(1-\frac{\overline{{\tau }_{w}}}{{\overline{{\tau }_{w}}}_{0}}\right).$$

Here, $\overline{{\tau }_{w}}$ is often referred to as the mean wall stress. Throughout this report, overbars indicate a long-time average, and ${\overline{{\tau }_{w}}}_{0}$ denotes the mean wall stress acting on the non-actuated, i.e., stationary, surface. While there are obvious practical obstacles to using oscillating elements on the surface of vehicles to reduce drag, the concept of spanwise control of the flow close to the surface clearly warrants closer examination.

For this actuation strategy, the mean wall stress has a functional dependence on the various actuation and flow parameters as:

3 $$\frac{\overline{{\tau }_{w}}}{\rho }={u}_{\tau }^{2}={g}_{1}({U}_{\infty },\ \delta ,\ \nu ,\ {\kappa }_{x},\ \omega ,A)$$

where *U*_*∞*_ is the freestream velocity, *δ* is the boundary layer thickness, *ν* is the fluid kinematic viscosity, and the friction velocity ${u}_{\tau }\equiv \sqrt{\overline{{\tau }_{w}}/\rho }$, *ρ* being the fluid density. The dimensional analysis gives:

4 $${{{{{{{\rm{DR}}}}}}}}={g}_{2}({\kappa }_{x}^{+},\ {T}_{{{{{{{{\rm{osc}}}}}}}}}^{+},\ {A}^{+},\ {{{{{{{{\mathrm{Re}}}}}}}}}_{\tau })$$

where ${\kappa }_{x}^{+}={\kappa }_{x}\ \nu \ /{{u}_{\tau }}_{0}$ (here ${{u}_{\tau }}_{0}\equiv \sqrt{{\overline{{\tau }_{w}}}_{0}/\rho }$), ${T}_{osc}^{+}=2\pi \ {{u}_{\tau }}_{0}^{2}\ /\ (\omega \nu )$, and ${A}^{+}=A/{{u}_{\tau }}_{0}$ are dimensionless actuation parameters. In our notation, the ‘+’ superscript refers to inner-scaled normalization based on the non-actuated flow using the viscous length scale ${\eta }_{v}\equiv \nu /{{u}_{\tau }}_{0}$ and friction velocity ${{u}_{\tau }}_{0}$. Hence, the friction Reynolds number ${{{{{{{{\mathrm{Re}}}}}}}}}_{\tau }=\delta {{u}_{\tau }}_{0}/\nu ={\delta }^{+}$. The Reynolds number is the ratio of inertial forces to viscous forces within a fluid flow; it also represents the range of scales of turbulent flow such that the higher the Reynolds number the broader the range of length and time scales involved. Specifically, ${{{{{{{{\mathrm{Re}}}}}}}}}_{\tau }$ can be interpreted as the ratio of a length scale representative of the large-scale motions (*δ*) to a length scale typical of the smallest-scale motions (*η*_*v*_).

To date, much of the attention has been paid to parameterizing how the drag reduction depends on the dimensionless actuation parameters: ${\kappa }_{x}^{+}$, *ω*^+^, and *A*^+^ (see Ricco et al.^19^). Full-scale simulations showing DR of 50%^13^ were all conducted at ${{{{{{{{\mathrm{Re}}}}}}}}}_{\tau }\le$ 1,000, significantly lower than the friction Reynolds numbers relevant for flows in aviation, pipelines, and wind turbines: typical values of ${{{{{{{{\mathrm{Re}}}}}}}}}_{\tau }$ are 4,000 on a wind turbine blade or in a long-distance oil pipeline, 6,000 mid-span on a Boeing 787 wing, and 10,000−100,000 along the length of a 787 fuselage during cruise. Experiments conducted to explore this drag-reduction approach have been similarly limited to low ${{{{{{{{\mathrm{Re}}}}}}}}}_{\tau }$^11,15^. Whether drag reduction persists at high ${{{{{{{{\mathrm{Re}}}}}}}}}_{\tau }$ has thus remained an unanswered question.

**Fig. 2:**
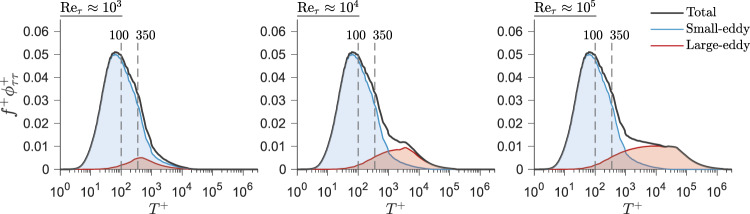
Wall stress spectrum dependence on Reynolds number. Pre-multiplied spectra of the wall stress *τ*_*w*_ with inner-scaling without actuation at ${{{{{{{{\mathrm{Re}}}}}}}}}_{\tau }=1{0}^{3},\ 1{0}^{4},$ and 10^5^ obtained from predictive models^20−22^. The blue-shaded region is the Reynolds number invariant small-scale contribution and the red-shaded region is the contribution from outer-region large-scale structures, which is dependent on Reynolds number. The abscissa for *T*^+^ = 100 and *T*^+^ = 350 are indicated on each of the plots by the vertical dashed lines to indicate the nominal peak intensity of the small-scale contribution and threshold between large- and small-scale contributions, respectively.

This question is important because the physics of drag reduction changes at high Reynolds numbers, in that the contribution of larger eddies to the wall stress grows with Reynolds number. Figure 2a−c shows the power spectral density (spectrum) of the fluctuating wall stress, *ϕ*_*ττ*_, for a non-actuated surface at ${{{{{{{{\mathrm{Re}}}}}}}}}_{\tau }=\,$10^3^, 10^4^, and 10^5^, respectively, as obtained from predictive models^20–22^ that are detailed in Supplementary Note 1 and Supplementary Fig. 1. The spectra are plotted in their premultiplied form, ${f}^{+}{\phi }_{\tau \tau }^{+}$ as a function of the non-dimensional time scale ${T}^{+}=1/{f}^{+}={{u}_{\tau }}_{0}^{2}/(f\nu )$, where *f* is frequency, so that the area under each spectrum corresponds to the variance of inner-scaled *τ*_*w*_, namely ${\overline{{\tau }_{w}^{2}}}^{+}$. That is, ${\overline{{\tau }_{w}^{2}}}^{+}=\int\nolimits_{0}^{\infty }{\phi }_{\tau \tau }^{+}\ {{{{{{{\rm{d}}}}}}}}{f}^{+}=\int\nolimits_{-\infty }^{\infty }{f}^{+}{\phi }_{\tau \tau }^{+}\ {{{{{{{\rm{d}}}}}}}}({{{{{{{\rm{ln}}}}}}}}\ {T}^{+})$. Each spectrum is decomposed into the contributions from high-frequency, ‘small-eddy’ motions and low-frequency, ‘large-eddy’ motions, shown as the blue- and red-shaded regions, respectively. Towards this, we have used the demarcation threshold of *T*^+^ = 350, following Mathis et al.^21^. Therefore, for simplicity, we refer to turbulent scales associated with *T*^+^ < 350 as ‘small-eddy’ and *T*^+^ > 350 as ‘large-eddy’.

We see that the small-eddy sub-component spectra feature a strong peak at *T*^+^ = 80−100, reflecting the intense high-frequency turbulence generated by the near-wall streaks (visible in Fig. 1b). Their contributions are Reynolds number invariant with inner-scaling and hence the spectra coincide for *T*^+^ ≲ 350^20,21^. However, unlike the small-eddy component, the contribution to the total wall-stress from the large-eddy sub-component increases with Reynolds number, from about 8% of the total variance at ${{{{{{{{\mathrm{Re}}}}}}}}}_{\tau }=1{0}^{3}$ to about 30% at ${{{{{{{{\mathrm{Re}}}}}}}}}_{\tau }=1{0}^{5}$. This trend indicates the increased superimposition and modulation of the energetic large-scale motions in the outer region of the boundary layer on the near-wall turbulence^20,23–26^.

At low Reynolds number, therefore, drag reduction approaches need to target the small eddies near the surface, because these eddies contribute most of the drag. However, at high Reynolds numbers, actuation targeting small-eddies could be extremely challenging as it typically requires very high actuation frequencies and wavenumbers^9,27,28^. Instead, an actuation scheme for high Reynolds numbers that couples to large-scale eddies might still achieve substantial drag reduction while also requiring much lower actuation frequencies.

To this end, we present the first experimental measurements of drag reduction induced by oscillatory surface actuation at high Reynolds numbers by installing a customized flow-control machine, called the surface-actuation test bed (SATB), in the large-scale turbulent boundary layer facility at the University of Melbourne. The SATB produces a discretized facsimile of the wall motion described by Eq. (1), i.e., a streamwise travelling wave, by oscillating 48 slats sinusoidally in the spanwise direction at frequencies up to 25 Hz (see Supplementary Movie 1). Each slat oscillates with a fixed half-stroke length, *d* = 18 mm, such that the spanwise velocity amplitude is *A* = *ωd*. The wavelength in the streamwise direction (*λ*) is approximately equal to the boundary layer thickness (*δ*) in order to maximize the likelihood that the actuation couples to the streamwise wavelength of the large-scale structures in the flow, which scale with *δ *^23,27^. The machine acts over an area of 2.4 m (streamwise) × 0.6 m (spanwise), or approximately 6 *δ* × 1.5 *δ* (see “Methods” section for details).

The friction drag was measured via two independent experimental techniques: directly with a large-scale drag balance (limited in this wind tunnel to 6,000 $\le {{{{{{{{\mathrm{Re}}}}}}}}}_{\tau }\le$ 12,800), and indirectly using a hot-wire anemometer (limited to 6,000 $\le {{{{{{{{\mathrm{Re}}}}}}}}}_{\tau }\le$ 9,700). For the drag-balance measurements, the SATB was mounted on a floating element that is effectively frictionless for streamwise displacements. A load cell directly measured the streamwise drag force acting on the entire exposed 3 m (streamwise) × 1 m (spanwise) floating element surface^29^, with and without actuation. For the hot-wire measurements, we determined the local wall stress (*τ*_*w*_) by acquiring a time series of streamwise velocity (*U*) at different wall-normal locations within the viscous sublayer, and over stationary and actuated surfaces. The velocity gradient at the surface was then estimated, and the wall stress derived through the relation *τ*_*w*_/*ρ* = *ν* d*U*/d*y* (see “Methods” section for details).

To isolate the trend of DR with ${{{{{{{{\mathrm{Re}}}}}}}}}_{\tau }$, it is essential to keep the dimensionless actuation parameters ${\kappa }_{x}^{+}$, ${T}_{{{{{{{{\rm{osc}}}}}}}}}^{+}$ and *A*^+^ fixed. However, the parameter space that can be explored in experiments are limited due to the combined operational envelope of the SATB and the wind tunnel. In experiments, ${A}^{+}=(2\pi /{T}_{{{{{{{{\rm{osc}}}}}}}}}^{+})(d/\delta ){{{{{{{{\mathrm{Re}}}}}}}}}_{\tau }$ and ${\kappa }_{x}^{+}=(2\pi /{{{{{{{{\mathrm{Re}}}}}}}}}_{\tau })(\delta /\lambda )$, where *d*/*δ* and *δ*/*λ* are approximately constant. Consequently, ${{{{{{{{\mathrm{Re}}}}}}}}}_{\tau }$ cannot be varied independent of $({A}^{+},\ {T}_{{{{{{{{\rm{osc}}}}}}}}}^{+},\ {\kappa }_{x}^{+})$. Further, the wind tunnel operation restricts the Reynolds numbers to ${{{{{{{{\mathrm{Re}}}}}}}}}_{\tau }\ge$ 6,000 (see “Methods” section). Therefore, to explore the Reynolds number dependencies of the drag reduction technique across a wider range, a series of large-eddy simulations (LES) were performed for ${{{{{{{{\mathrm{Re}}}}}}}}}_{\tau }\le$ 2,000. In both the experiments and LES, the streamwise travelling wave always moved in the upstream direction.

## Results and discussion

### Drag reduction dependence on ${{{{{{{{\mathrm{Re}}}}}}}}}_{\tau }$

**Fig. 3:**
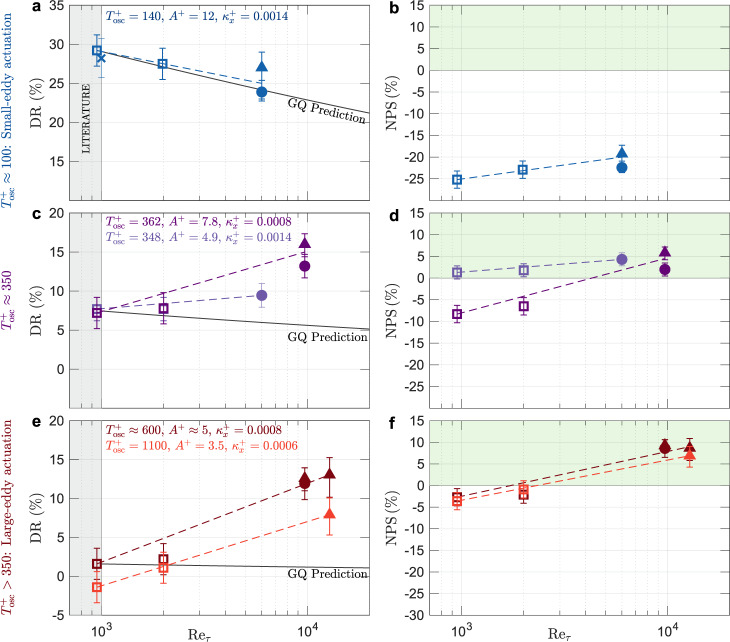
Drag reduction and net power savings increase with friction Reynolds number far above predicted values when oscillatory surface actuation couples to large eddies. DR(%) and NPS(%) for small-eddy actuation (**a**, **b**) confirming an ${{{{{{{{\mathrm{Re}}}}}}}}}_{\tau }$ trend of DR predicted by the formulation of Gatti and Quadrio^13,GQ^ and resulting in negative NPS. In contrast, large-eddy actuation (**e**, **f**) leads to DR increase at higher ${{{{{{{{\mathrm{Re}}}}}}}}}_{\tau }$ (dark red) to 10−15%, ~ 10-fold greater than predicted and resulting in positive NPS ≈ 10%. **c**, **d** Trends of DR(%) and NPS(%) for an intermediate actuation corresponding to the large-scale cut-off ${T}_{{{{{{{{\mathrm{osc}}}}}}}}}^{+}\approx 350$. Open squares indicate large-eddy simulations (LES), circles indicate hot-wire anemometry experiments, triangles indicate drag balance experiments and crosses indicate direct-numerical simulation (DNS) result of Gatti and Quadrio^13^. Error bars indicate one standard deviation estimated uncertainty ranges. Dashed lines connecting experimental and computational measurements are included as a visual aid only. See Supplementary Table 1 for details of the flow and actuation.

The drag reduction results, shown in Fig. 3 as a function of Reynolds number, are the first ever reported in experiments or full-scale numerical simulations for ${{{{{{{{\mathrm{Re}}}}}}}}}_{\tau } >$ 1,000. The results are parsed into three actuation categories: small-eddy, large-eddy, and intermediate. Following the decomposition in Fig. 2, small-eddy and large-eddy actuation refer to actuation on time scales typical of the smaller, near-wall motions (${T}_{{{{{{{{\rm{osc}}}}}}}}}^{+}\approx 100$) and the larger, outer-region motions (${T}_{{{{{{{{\rm{osc}}}}}}}}}^{+} > 350$), respectively. The intermediate category is actuation where the oscillation period is comparable to the large-scale cut-off (${T}_{{{{{{{{\rm{osc}}}}}}}}}^{+}\approx 350$).

In addition to the drag reduction results, we also present the net power saving (NPS), computed according to refs. ^30^ and ^31^ (see Supplementary Note 2 and Supplementary Fig. 2 for details). This quantity measures the relative change in the total power cost between an oscillating wall and its stationary counterpart for an idealized actuator. Therefore, a positive NPS implies that the drag reduction due to the actuation outweighs the cost of input power required for the actuation and vice versa.

Under small-eddy actuation, actuation parameters were selected to be nominally optimal for controlling the near-wall motions, viz. *A*^+^ = 12, ${T}_{{{{{{{{\rm{osc}}}}}}}}}^{+}=140$ and ${\kappa }_{x}^{+}=0.0014$^7,13^. At ${{{{{{{{\mathrm{Re}}}}}}}}}_{\tau }=$ 6,000 we measured DR ≈ 25% – only a modest decrease from the 30% value seen at ${{{{{{{{\mathrm{Re}}}}}}}}}_{\tau }=$ 951 (Fig. 3a). This trend of decreasing DR with increasing ${{{{{{{{\mathrm{Re}}}}}}}}}_{\tau }$ has been extensively discussed in the literature^19^ and in Fig. 3a we see that the predictive model of Gatti & Quadrio^13^ works well in estimating this trend. The GQ model adopts a fixed shift in the inner-scaled mean velocity profile due to actuation, which effectively assumes that DR depends only on the actuation parameters that are inner-scaled with the local wall stress at a given Reynolds number. Accordingly, the model is seen to work well for small-eddy actuation, which targets the inner-scaled motions, but it breaks down for actuation schemes that target the larger-scale motions, which do not follow inner scaling—see Fig. 3c, e, to be discussed shortly.

The results in Fig. 3a confirm that, in principle, small-eddy actuation could offer an effective drag reduction strategy even at high Reynolds numbers. As we can see in Fig. 3b, however, this level of drag reduction can come at a substantial cost in power: the actuation incurs a net power penalty between −27 and −20% for 951 $\le {{{{{{{{\mathrm{Re}}}}}}}}}_{\tau }\le$ 6,000. Previous numerical studies^13,19,31^ similarly report negative NPS with small-scale actuation and an upstream travelling wave.

We next consider the data for two sets of large-eddy actuation parameters where ${T}_{{{{{{{{\rm{osc}}}}}}}}}^{+} > 350$ (Fig. 3e, f). For the first set *A*^+^ = 4.6, ${T}_{{{{{{{{\rm{osc}}}}}}}}}^{+}=604$, ${\kappa }_{x}^{+}=0.0008$, and for the second set *A*^+^ = 3.5, ${T}_{{{{{{{{\rm{osc}}}}}}}}}^{+}=1100$, ${\kappa }_{x}^{+}=0.0006$. As noted, these parameters could not be chosen at will as they are constrained by the operation of the SATB and the wind tunnel. For the large-eddy (low-frequency) actuation parameters, the LES predicts that actuation with the first set of parameters (Fig. 3e, dark red symbols) results in DR = 1.6% at ${{{{{{{{\mathrm{Re}}}}}}}}}_{\tau }=$ 951. This result agrees with the extrapolation schemes based on low-${{{{{{{{\mathrm{Re}}}}}}}}}_{\tau }$ studies, which predict little or no drag reduction under these conditions. Under the same operating conditions at ${{{{{{{{\mathrm{Re}}}}}}}}}_{\tau }=$ 12,800, however, we measured DR = 13%, at least an order of magnitude higher than extrapolation from prior studies would predict. We thus conclude that large-eddy actuation can lead to substantial drag reduction. Moreover, this level of drag reduction increases with Reynolds number. For the second set of actuation parameters, with an even longer period of oscillation and lower amplitude (Fig. 3e, light red symbols), a similar trend is seen, with DR = −1.4% at ${{{{{{{{\mathrm{Re}}}}}}}}}_{\tau }=$ 951 but DR = 8% at ${{{{{{{{\mathrm{Re}}}}}}}}}_{\tau }=$ 12,800.

The discovery that drag reduction increases with Reynolds number in response to large-eddy actuation indicates that this control method engages a drag reduction pathway that low-${{{{{{{{\mathrm{Re}}}}}}}}}_{\tau }$ models neither capture nor predict. In applications where only low-frequency actuation is practical, this pathway may enable additional drag reduction that improves efficiency and/or performance. In particular, for any oscillating surface actuation the power needed to control a spanwise-driven Stokes layer is proportional to *ω*^5/2 ^^30^, which in turn scales as ${({{{{{{{{\mathrm{Re}}}}}}}}}_{\tau }{{u}_{\tau }}_{0}/\delta )}^{5/2}$ for actuation frequencies that match those of the near-wall small eddies. At high Reynolds numbers, the considerably lower frequencies needed for large-eddy actuation compared to small-eddy actuation, therefore, results in significantly reduced power costs. For example, at ${{{{{{{{\mathrm{Re}}}}}}}}}_{\tau }=$ 12,800 in the wind tunnel, as ${T}_{{{{{{{{\rm{osc}}}}}}}}}^{+}$ increases from 100 to 1,000, the corresponding frequency of oscillation drops from 150 to 15 Hz, greatly reducing actuation power while still delivering a drag reduction of about 8%. The consequences of this reduced input power are shown in the Fig. 3f, where the net power savings in each case is estimated to go from −4% at ${{{{{{{{\mathrm{Re}}}}}}}}}_{\tau }=$ 951 up to ≈ +10% at ${{{{{{{{\mathrm{Re}}}}}}}}}_{\tau }=$ 9,700. In other words, at ${{{{{{{{\mathrm{Re}}}}}}}}}_{\tau }=9,700$ the total power cost to oscillate the wall and pump the flow over it is 10% less than the power cost to pump the flow over a stationary wall.

Finally, we consider the data for actuation at two intermediate frequencies corresponding to ${T}_{{{{{{{{\rm{osc}}}}}}}}}^{+}\approx 350$ (Fig. 3c, d). For this actuation, the trends of DR and NPS lie between that of small-eddy and large-eddy actuation. At ${{{{{{{{\mathrm{Re}}}}}}}}}_{\tau }\le 2000$, DR ≈ 8% for both sets of actuation parameters. As ${{{{{{{{\mathrm{Re}}}}}}}}}_{\tau }$ increased to 9,700, the drag reduction for the first set of actuation parameters (*A*^+^ = 7.8, dark purple symbols) increased to a level between 13 and 16%. While these actuation parameters incur a net power cost at ${{{{{{{{\mathrm{Re}}}}}}}}}_{\tau }\le$ 2,000 (NPS ≈ − 8%), they are able to generate NPS = 2−6% at ${{{{{{{{\mathrm{Re}}}}}}}}}_{\tau }=$ 9,700. The second set of intermediate actuation parameters using a lower oscillation amplitude (*A*^+^ = 4.9, light purple symbols) was similarly able to generate DR = 9.5% drag reduction at ${{{{{{{{\mathrm{Re}}}}}}}}}_{\tau }=$ 6,000 while maintaining NPS = 1−5% for 951 $\le {{{{{{{{\mathrm{Re}}}}}}}}}_{\tau }\le 6,000$.

### Effect of actuation on near-wall turbulence

**Fig. 4:**
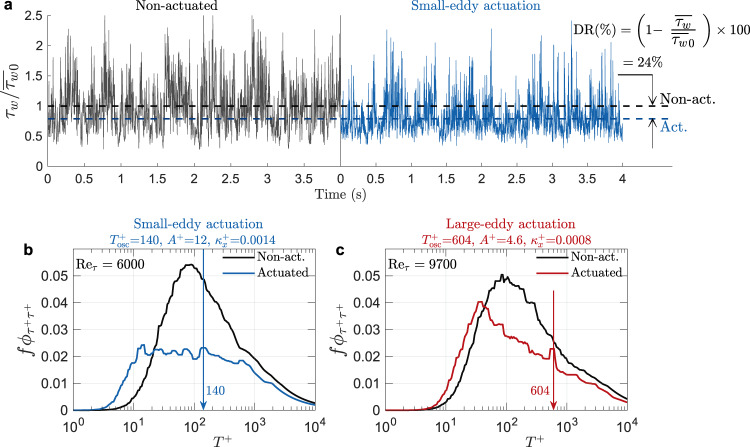
Temporal fluctuations in wall stress signals and their spectra illustrate the effects of wall actuation. **a** Sample of time-series measurements of the wall stress *τ*_*w*_ at ${{{{{{{{\mathrm{Re}}}}}}}}}_{\tau }=$ 6,000 without actuation and with small-eddy actuation (${A}^{+}=12,\ {T}_{{{{{{{{\rm{osc}}}}}}}}}^{+}=140,\ {\kappa }_{x}^{+}=0.0014$), shown in solid black and blue lines, respectively. Mean values of each signal are denoted by dashed lines, whose difference indicates that the wall oscillation results in DR = 24%. **b** Spectra of *τ*_*w*_ at ${{{{{{{{\mathrm{Re}}}}}}}}}_{\tau }=$ 6,000 with and without small-eddy actuation with same parameters as in (**a**). **c** Spectra of *τ*_*w*_ at ${{{{{{{{\mathrm{Re}}}}}}}}}_{\tau }=$ 9,700 with and without large-eddy actuation (${A}^{+}=4.6,\ {T}_{{{{{{{{\mathrm{osc}}}}}}}}}^{+}=604,\ {\kappa }_{x}^{+}=0.0008$), shown in red and black lines, respectively. Spectra in (**b**, **c**) are obtained using hot-wires with 7.5 ≤ *l*^+^ ≤ 12 for $6000\le {{{{{{{{\mathrm{Re}}}}}}}}}_{\tau }\le 9700$, where *l* is the length of the hot-wire sensor.

The efficacy of small-eddy and large-eddy actuation can be traced to the underlying physics of the frequency-specific turbulent motions that contribute to skin-friction drag at high Reynolds numbers. When we examine the time-series measurements of the wall stress at a Reynolds number of 6,000 (Fig. 4a), we see that the wall stress fluctuations contain a broad frequency content. A small-eddy actuation using nominally optimal parameters [${A}^{+}=12,\ {T}_{{{{{{{{\rm{osc}}}}}}}}}^{+}=140,\ {\kappa }_{x}^{+}=0.0014$], resulting in DR = 24%, is observed to both decrease the mean wall stress and attenuate its fluctuations. To understand the scale-specific effect of actuation, we compare the corresponding spectra of the fluctuating wall stress, *ϕ*_*ττ*_, for the non-actuated and actuated cases, shown in Fig. 4b. For the non-actuated case, as seen earlier in Fig. 2, a strong peak in the spectrum is located at *T*^+^ ≈ 100, which reflects the intense high-frequency turbulence generated by the near-wall streaks. The small-eddy actuation is observed to severely attenuate all scales for *T*^+^ < 1,000, while shifting the spectrum towards lower time scales.

Figure 4c shows the same comparison for one of the large-eddy actuation sets used [${A}^{+}\approx 5,\ {T}_{{{{{{{{\rm{osc}}}}}}}}}^{+}\approx 600,\ {\kappa }_{x}^{+}=0.0008$] at ${{{{{{{{\mathrm{Re}}}}}}}}}_{\tau }=$ 9,700 where DR ≈ 13% (Fig. 3e). Here, the actuation is seen to significantly suppress the contribution from motions having 50 ≲ *T*^+^ ≲ 2,000, whereas high-frequency motions of *T*^+^ ≲ 50 are virtually unaffected. Notably, the attenuating effects of large-eddy actuation on wall stress fluctuations span a broad range of scales and are not confined to the large-scale contributions. This feature, combined with substantial DR (Fig. 3e), draws us to conclude that large-eddy actuation becomes an alternative effective pathway for suppressing the wall stress at higher Reynolds numbers, where the contributions to the wall stress at larger time scales and lower frequencies increases. Moreover, this pathway promises net power savings making it potentially attractive for future applications.

**Fig. 5:**
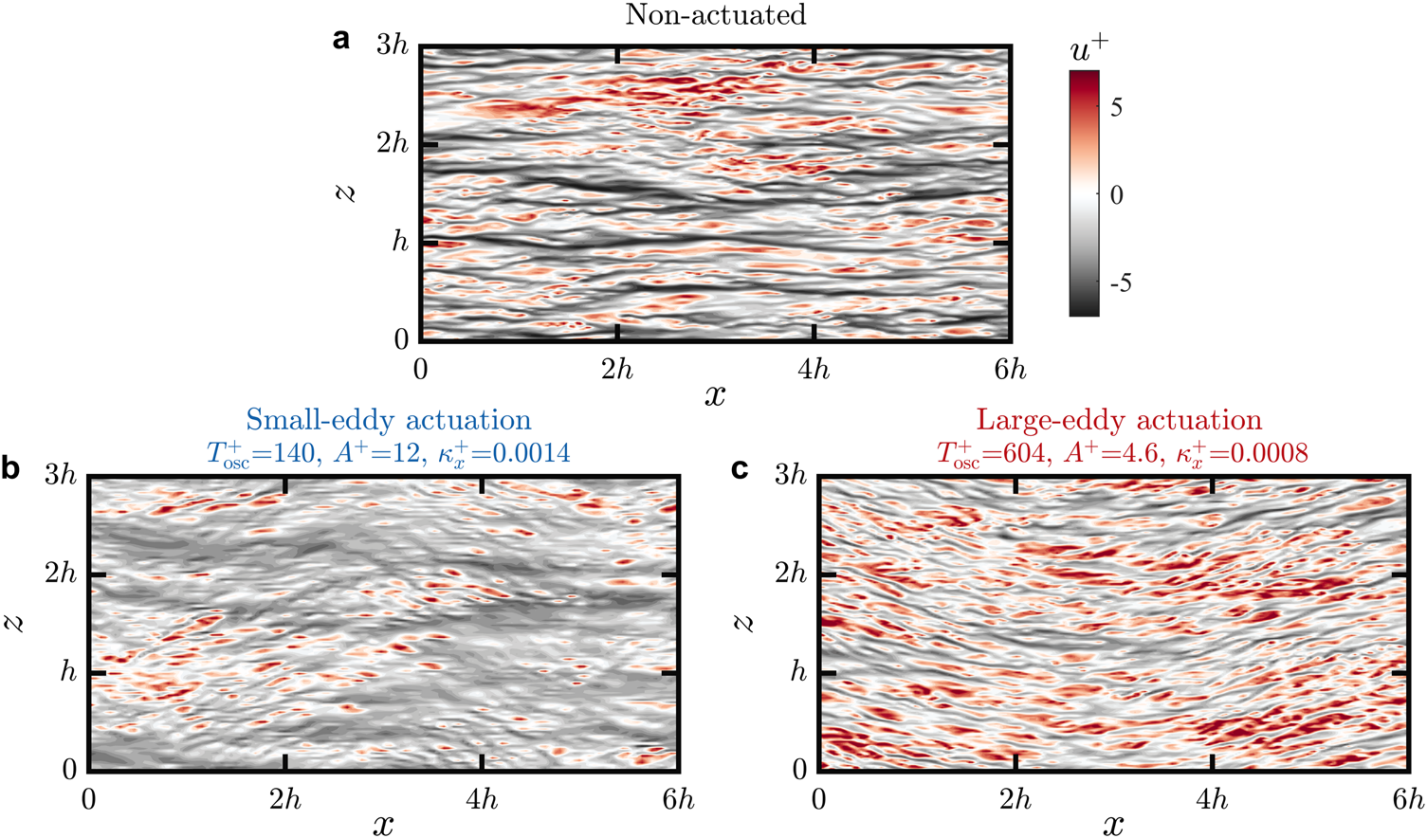
Visualizations of near-wall turbulence illustrate the effects of actuation at low ${{{{{{{{\mathrm{Re}}}}}}}}}_{\tau }$. Contours of streamwise velocity fluctuations (*u*) from LES at ${{{{{{{{\mathrm{Re}}}}}}}}}_{\tau }=$ 951 and at *y*^+^ ≈ 12 for **a** no actuation, **b** small-eddy actuation, and **c** large-eddy actuation matching the actuation parameters shown in Fig. 4b, c at high ${{{{{{{{\mathrm{Re}}}}}}}}}_{\tau }$. *h* is the height of the open channel in the LES.

It is interesting to compare the effects, on the turbulence, of using the same small-eddy and large-eddy actuation parameters at low Reynolds numbers with those at high Reynolds number shown in Fig. 4b, c. This is demonstrated in Fig. 5 for the LES results at ${{{{{{{{\mathrm{Re}}}}}}}}}_{\tau }=$ 951 using visualizations of the flow near the surface. Figure 5a, b clearly shows that small-eddy actuation depletes the strength of the streaks, thereby significantly reducing the intensity of turbulence near the surface. This result is consistent with the attenuation of higher Reynolds number spectra in Fig. 4b for the same small-eddy actuation, and therefore we conclude that the strategy of using small-eddy actuation to disrupt the streaks effectively produces substantial drag reduction at all Reynolds numbers. Large-eddy actuation at low ${{{{{{{{\mathrm{Re}}}}}}}}}_{\tau }$, however, is not effective at breaking up the near-wall motions and attenuating their intensity, as visualized in Fig. 5c. Instead, the streaks simply meander passively in response to the actuation, following the travelling wave at the surface. This behaviour is to be expected at this low Reynolds number where the turbulent scales that match the time period of the large-eddy actuation (${T}_{{{{{{{{\rm{osc}}}}}}}}}^{+}=600$) carry very little energy (see Fig. 2a).

These results highlight how important it is to understand and leverage the physics at large scales in order to achieve significant drag reduction in turbulent flows at high Reynolds numbers.

Finally, we note that our focus on total drag reduction and the physical pathways that produce these results opens new avenues for practical applications. Although implementing this effect on vehicles or in pipelines to reduce drag will require appreciable innovation, the concept of introducing spanwise disturbances into the near-surface flow to reduce drag holds great promise. Future engineering strategies may exploit the combination of increasing efficacy at high Reynolds numbers and the dramatically lower input power requirements of large-eddy actuation, particularly in applications where only low-frequency actuation would be practical.

## Methods

### High Reynolds number boundary layer wind tunnel

Experiments were performed in the high Reynolds number boundary layer wind tunnel facility at the University of Melbourne. The tunnel test section is over 27 m long with a 2 × 1 m cross-section. The flow is tripped at the entrance to the working section by a 35 mm wide stripe of P40 grit sand paper (425–500 μm grit size). A zero-pressure gradient condition is maintained throughout the working section by bleeding the top wall boundary layer, resulting in a constant free-stream mean velocity along the entire working section maintained to within ±0.5% and free-stream turbulence intensities (*u*_rms_/*U*_*∞*_) < 0.2% for all locations and test conditions evaluated here. All current experiments are performed on the tunnel floor at a streamwise location of *x* ≈ 21 m, where the turbulent boundary layer thickness is *δ* = 0.385−0.39 m, with friction Reynolds numbers between $6,000\le {{{{{{{{\mathrm{Re}}}}}}}}}_{\tau }\le 13,000$ for 7 m/s ≤ *U*_*∞*_ ≤ 15 m/s (see Supplementary Table 1). Additional details of the facility and its validation can be found in ref. ^29^.

### Surface actuation test bed

A unique actuation system, dubbed the surface actuation test bed or SATB, was custom designed and built to actuate the high-Reynolds number boundary layer with low-frequency spanwise wall oscillations. The SATB, shown in Supplementary Fig. 3, actuates the overlying flow with a series of forty-eight, 50 mm-wide slats that oscillate sinusoidally in time in the spanwise direction with a fixed half-stroke length, *d* = 18 mm, and a maximum spanwise velocity *A* = *ωd*. The slat oscillation is driven by four independently controllable machines that are run synchronously along the length of the floating element. Each machine has 12 slats whose phase is controlled by a central camshaft. The sinusoidal travelling wave is discretized with six slats constituting a fixed streamwise wavelength *λ* = 2*π*/*κ*_*x*_ = 0.3 m^11^. The four machines are driven by servomotors with oscillation frequencies up to *f* = *ω*/(2*π*) = 25 Hz and are phase synchronized to ensure a continuous 8*λ* long upstream travelling wave of spanwise velocity. A video showing the SATB in operation is included as Supplementary Movie 1.

The high degree of precision and tolerancing associated with the fabrication of the machine allows us to achieve a close facsimile of sinusoidal spanwise motion. Gaps between individual slats are ${{{{{{{\mathcal{O}}}}}}}}(100\ \upmu {{{{{{{\rm{m}}}}}}}})$. A chamber below the floating element was pressurized to the static pressure in the tunnel working section to mitigate any flow leakage through the gaps between the slats. The flatness and vertical displacement of the slats along SATB during oscillation were measured with a laser displacement sensor (Keyence LK-031, ±1 μm accuracy, 30 μm beam diameter). The maximum step height between slats and the peak to peak displacement during oscillations were typically ${{{{{{{\mathcal{O}}}}}}}}(10\ \upmu {{{{{{{\rm{m}}}}}}}})$ for most slats and ${{{{{{{\mathcal{O}}}}}}}}(100\ \upmu {{{{{{{\rm{m}}}}}}}})$ in the worst case. Further, the measurements obtained over the stationary SATB were found to agree well with the equivalent smooth wall measurements previously obtained in the facility^29^. Apart from the absence of surface motion, no surface modifications were applied between the actuated and non-actuated cases.

### Drag balance

An area-averaged drag measurement from the actuation was obtained by mounting the actuation system on a large-scale floating element. The exposed 3 × 1 m surface of the floating element is approximately 21 m downstream from the boundary layer trip, flush with the tunnel floor, and centred between the side walls of the working section. A series of air bearings are used to restrict the spanwise motion of the balance while allowing for nearly frictionless movement in the streamwise direction. Streamwise displacement of the floating element is constrained by a 6 N load cell (0.06% accuracy full scale) which measures the average streamwise drag on the total exposed surface of the floating element from the passing flow. The signal from the load cell was sampled for at least 60 s at 1000 Hz. Centred within the floating element surface is a rectangular cutout of 2.7 × 0.7 m^2^, where the surface actuation test bed (SATB) is flush-mounted. In total, approximately 48% of the entire floating element surface was actuated with surface oscillations with an additional 8% of floating element surface located immediately downstream of the actuator where latent drag reduction effects could be present. The drag reduction was computed by comparing the drag force measured by the calibrated load cell with and without the actuation. Further details on the construction, calibration, and validation of the floating element system can be found in ref. ^29^.

### Hot-wire anemometry

Local streamwise wall-shear stress measurements were obtained using hot-wire anemometry in close proximity to the wall (both stationary and actuated). For a given location on the wall, the instantaneous, streamwise wall stress on the surface, *τ*_*w*_, is given by

5 $$\frac{{\tau }_{w}}{\rho }={\left.\nu \frac{\partial U(y)}{\partial y}\right|}_{y = 0}.$$

For a turbulent wall-bounded flow, the velocity gradient at the wall surface can be approximated to within a few percent error^32^ by measuring the streamwise velocity in close proximity to the wall where

6 $$\frac{{\tau }_{w}}{\rho }=\nu \frac{U(y)}{y}+{{{{{{{\mathcal{O}}}}}}}}({y}^{2})\ :\ {y}^{+}\le 10.$$

Using this relationship, we determined *τ*_*w*_ by measuring the streamwise velocity at two to four different wall-normal locations within the viscous sublayer. To determine the drag reduction, the measurements were made for the non-actuated and actuated cases, where for the latter, hot-wire signals were acquired over the actuated surface. The hot-wire results shown in Fig. 4b, c were obtained in the viscous sublayer, corresponding to *y*^+^ ≤ 5 (*y* = 370 and 200 μm for the ${{{{{{{{\mathrm{Re}}}}}}}}}_{\tau }=6,000$ and 9,700 cases, respectively). Representative hot-wire profiles of mean streamwise velocity and variance are shown in Supplementary Fig. 4. The non-actuated and actuated mean velocity profiles collapse onto the DNS data^33^ when normalized with the local friction velocity. Here, ${\overline{U}}^{* }=\overline{U}/{u}_{\tau }$; ${\overline{U}}^{+}=\overline{U}/{{u}_{\tau }}_{0}$, etc.

The hot-wire sensors were fabricated from Wollaston wires that were soldered to the Dantec boundary layer type miniature prongs. The wires were etched to reveal a 0.5 mm long, 2.5 μm diameter platinum sensing element. The probes were operated with an in-house Melbourne University Constant Temperature Anemometer (MUCTA) circuit at an overheat ratio of 1.8. The resulting frequency response was 20 kHz based on the −3 dB cutoff as verified using a square-wave electronic test. The probe response was calibrated in the tunnel freestream at 15 different velocities and fitted with a third-order polynomial. The hot-wire probe was positioned in close proximity to the wall using a stepper motor-driven vertical traverse equipped with a linear optical encoder (RENISHAW RGH24-type, ±0.5 μm accuracy). The absolute displacement of the probe measurement was validated using a depth measuring displacement microscope (Titan Tool Supply, ±1 μm accuracy). The signals were sampled at 40 and 50 kHz, respectively, at ${{{{{{{{\mathrm{Re}}}}}}}}}_{\tau }=6,000$ and 9,700, and low-pass filtered using an 8-pole Butterworth filter (Frequency Devices, Inc. model 9002) with the roll-off frequency set at half the sampling frequency to minimize aliasing. To ensure converged statistics, the signals were sampled for *t* = 60−90 s in any case, such that the non-dimensional boundary layer turnover times (*tU*_*∞*_/*δ*), associated with the largest structures in the flow, are 1100−2500.

### Computational method

Our computational approach is well-resolved large-eddy simulation (LES), with the dynamic Smagorinsky^34,35^ as the subgrid-scale model. The flow scales are resolved down to the viscous sublayer at the bottom wall (no wall modelling). The solver is a fourth-order accurate finite-difference code^36,37^. The domain is an open channel with the size (*L*_*x*_, *L*_*y*_, *L*_*z*_) ≃ (6*h*, *h*, 3*h*), and with periodic boundary conditions in the streamwise and spanwise directions. The boundary conditions at the bottom wall are *u* = *v* = 0 and $w(x,z,t)=A\sin ({k}_{x}x-\omega t)$, and at the top boundary are free-slip and impermeable conditions (∂*u*/∂*y* = ∂*w*/∂*y* = *v* = 0). The calculations are performed at friction Reynolds numbers ${{{{{{{{\mathrm{Re}}}}}}}}}_{\tau }=951$ and 2003 and the details of the grid are provided in Supplementary Table 2. For each set of dimensionless actuation parameters $[{A}^{+},{T}_{{{{{{{{\rm{osc}}}}}}}}}^{+},{\kappa }_{x}^{+}]$, we perform grid convergence tests until the change in the percent drag reduction DR(%) is less than 2%. We further validate our LES setup by comparison with the direct numerical simulation (DNS) of Gatti & Quadrio^13^ where data is available.

## Supplementary information

Supplementary Information

Description of Additional Supplementary Files

Supplementary Movie 1

## Data Availability

The datasets generated during the current study are available in the University of Melbourne Figshare repository, 10.26188/16557060^38^.

## Code availability

The code to support the findings in this work is available upon request from the authors.
