# Supplementary material for: An energy-efficient pathway to turbulent drag reduction
Source: Nat Commun. 2021 Oct 4;12:5805. doi: 10.1038/s41467-021-26128-8 (PMC8490469; doi:10.1038/s41467-021-26128-8)
Supplement: Supplementary file 1 — Supplementary Information [file 41467_2021_26128_MOESM1_ESM.pdf]

Supplementary Information

An energy-efficient pathway to turbulent drag reduction

Ivan Marusic,[1*] Dileep Chandran,[1] Amirreza Rouhi,[2] Matt K. Fu,[3]
David Wine,[4] Brian Holloway[4], Daniel Chung,[1] and Alexander J. Smits[5]

[1]Dept. of Mechanical Engineering, University of Melbourne, VIC 3010, AU
[2]Dept. of Engineering, School of Science and Technology, Nottingham Trent University, Nottingham NG11 8NS, UK
[3]Graduate Aerospace Laboratories (GALCIT), Caltech, Pasadena, CA 91125, USA
[4]Intellectual Ventures, Bellevue, WA 98005, USA
[5]Dept. of Mechanical and Aerospace Engineering, Princeton University, Princeton, NJ 08544, USA

[*]Correspondence should be addressed to imarusic@unimelb.edu.au.

Supplementary Note 1. Predictive model for wall shear stress spectra

The spectra of fluctuating wall shear stress plotted in Fig. 2 of the manuscript are obtained from the predictive models (*1–3*). As highlighted in Supplementary Fig. 1, the total inner-scaled wall stress spectrum is obtained as the summation of contributions from the small-eddy motions (blue-shaded) and the outer region, large-eddy motions (red-shaded). The former is the Reynolds number invariant universal signal of Mathis et al. (*2*) that is obtained by high-pass filtering the empirical wall shear stress signal at a cut-off frequency of $f^+ = 2.65 \times 10^{-3}$ ($T^+ \approx 350$). The red-shaded large-eddy spectra, however, are obtained from a phenomenological model (*3*) where the energetic large-scale motions in the outer region that contribute

to the wall stress are modeled using hierarchies of eddies that are 'attached' to the wall and superstructure/VLSM contributions. The magnitude of their contribution at the wall is modeled as a constant fraction of their energy in the outer region. Supplementary Fig. 1b, shows the variation of wall stress intensity (area under the total spectra) with Reynolds number. The predictions from the model compare well with the DNS data at low Reynolds numbers and the wall stress intensity is observed to increase as $\ln(\mathrm{Re}_\tau)$ due to the increased contribution from the large-eddies.

Supplementary Note 2. Power calculation

The input power required to operate our actuation mechanism is:

$$P_{\mathrm{in}}^+ = \frac{1}{T_{\mathrm{avg}}^+ L_x^+ L_z^+} \int_{t^+}^{t^+ + T_{\mathrm{avg}}^+} \int_0^{L_x^+} \int_0^{L_z^+} {w_s}^+ \left(\frac{\partial w^+}{\partial y^+}\bigg|_{y^+=0} \right) dx^+ dz^+ dt^+ \tag{1}$$

where P_{in}^+ is the input power, $T_{\mathrm{avg}}^+, L_x^+, L_z^+$ are the averaging time, streamwise and spanwise domain lengths, respectively; ${w_s}^+ \equiv w^+|_{y^+=0}$, $\partial w^+/\partial y^+|_{y^+=0}$ are the instantaneous spanwise velocity and its wall-normal gradient at the wall ($y^+ = 0$), respectively. The quantities with superscript "+" are normalised by ν and $u_{\tau 0}$ of the non-actuated (stationary) case.

The net power saving (NPS) measures the difference between the total power cost for the non-actuated case (P_0^+) and the oscillating case ($P^+ + P_{\mathrm{in}}^+$)

$$\mathrm{NPS} = \frac{P_0^+ - (P^+ + P_{\mathrm{in}}^+)}{P_0^+} = \mathrm{DR} - \frac{P_{\mathrm{in}}^+}{P_0^+} \tag{2}$$

where P_0^+ and P^+ are the pumping power required to drive the flow over the stationary wall and oscillating wall, respectively; P_0^+ and P^+ can be computed from the bulk velocity of the stationary case $U_{b_0}^+$, i.e. $P_0^+ = U_{b_0}^+$ and $P^+ = (1 - \mathrm{DR})U_{b_0}^+$.

Calculating the integral in equation (3) is possible with LES. However, for the experiments we do not have the instantaneous spanwise velocity gradient at the wall, $\partial w^+/\partial y^+|_{y^+=0}$. Instead, we use the laminar generalized Stokes layer (GSL) theory by Quadrio & Ricco (*4*). This

theory provides the instantaneous spanwise velocity profile

$$w^+(x^+, y^+, t^+) = \\ A^+\mathcal{R}\left\{Ce^{i(k_x^+ x^+ - \omega^+ t^+)}\text{Ai}\left[e^{\pi i/6}\left(k_x^+[1-\text{DR}]\right)^{1/3}\left(y^+ - \frac{\omega^+}{k_x^+[1-\text{DR}]} - \frac{ik_x^+}{1-\text{DR}}\right)\right]\right\} \quad (3)$$

where $C = \left\{\text{Ai}\left[ie^{i\pi/3}\left(k_x^+\left[1-\text{DR}\right]\right)^{1/3}(\omega^+/k_x^+ + ik_x^+)/[1-\text{DR}]\right]\right\}^{-1}$, Ai is Airy function of the first kind, and $\mathcal{R}\{...\}$ is the real part of the argument. Gatti & Quadrio (*5*) compared P_{in}^+/P_0^+ calculated using w^+ from their DNS to using the GSL theory (equation 5) and obtained excellent agreement between the two methods. In Supplementary Fig. 2, we repeat the same comparison between our LES data and the GSL theory. We compare the input power ratio P_{in}^+/P_0^+ for all the LES cases that we report in Fig. 3 of the manuscript. We obtain good agreement between LES and the GSL theory. For the large-eddy actuation cases, the maximum difference is less than 1%, and for the small-eddy actuation cases the maximum difference is less than 3%.

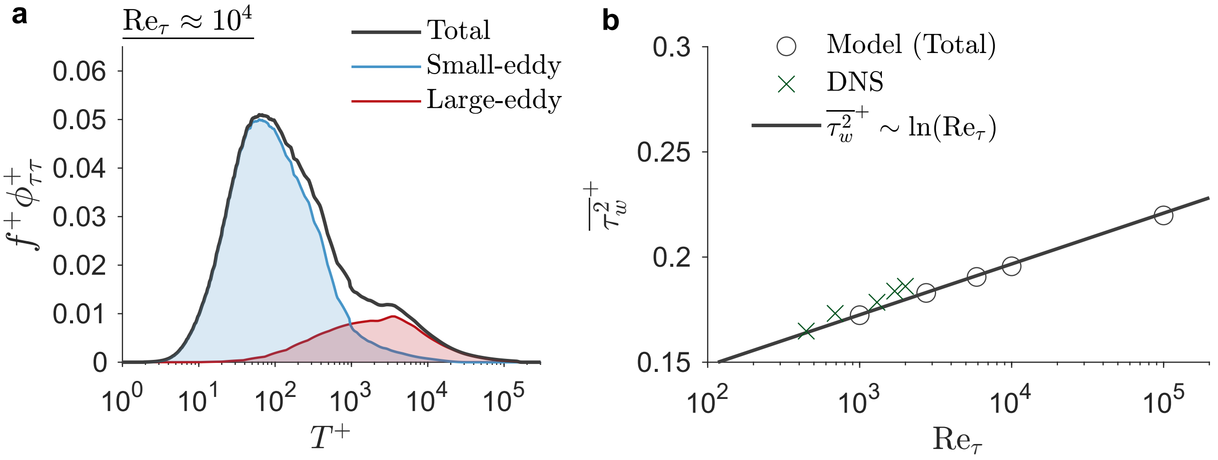

Supplementary Fig. 1. **Predictive model for wall shear stress spectra.** (**a**) Pre-multiplied spectra of the wall stress τ_w without actuation obtained from predictive models (*1–3*). The blue-shaded region is the Reynolds number invariant small-scale contribution and the red-shaded region is the contribution from outer-region large-scale structures which is dependent on Reynolds number. (**b**) Variance of wall stress fluctuations versus Re_τ. The green crosses are the DNS data of (*6, 7*). The solid line indicates logarithmic trend of $\overline{\tau_w^2}^+$ with Re_τ.

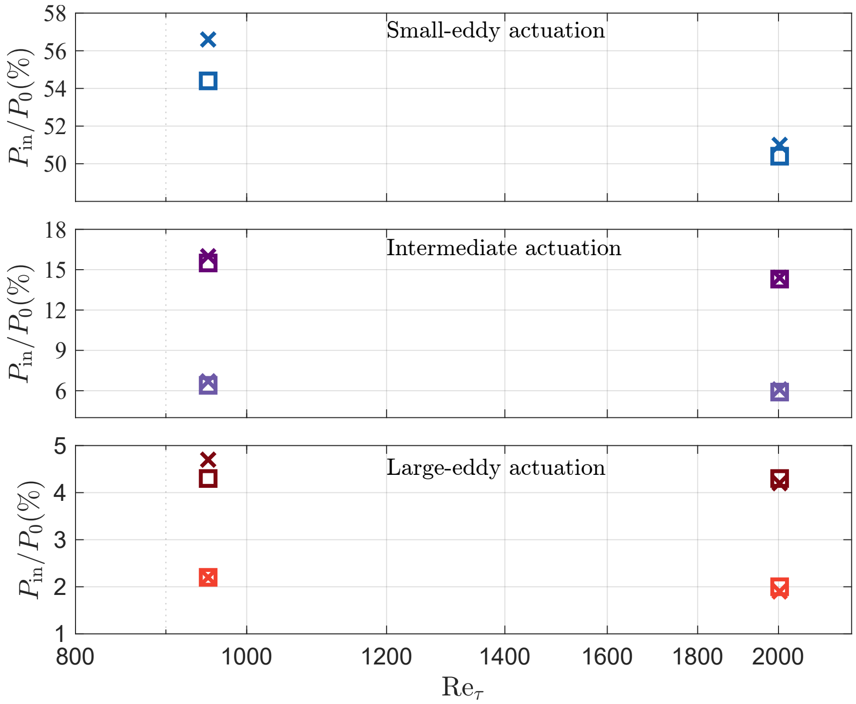

Supplementary Fig. 2. **Comparison of the input power ratio obtained from LES and GSL.** Input power ratio, P_{in}^+/P_0^+, obtained from LES (square symbols) versus generalised Stokes layer theory (cross symbols). P_{in}^+ (equation 3) is the required input power to oscillate the wall, and P_0^+ is the required pumping power to drive the flow over the stationary wall. We perform this comparison for all the ten LES cases that we report in Fig. 3 in the manuscript.

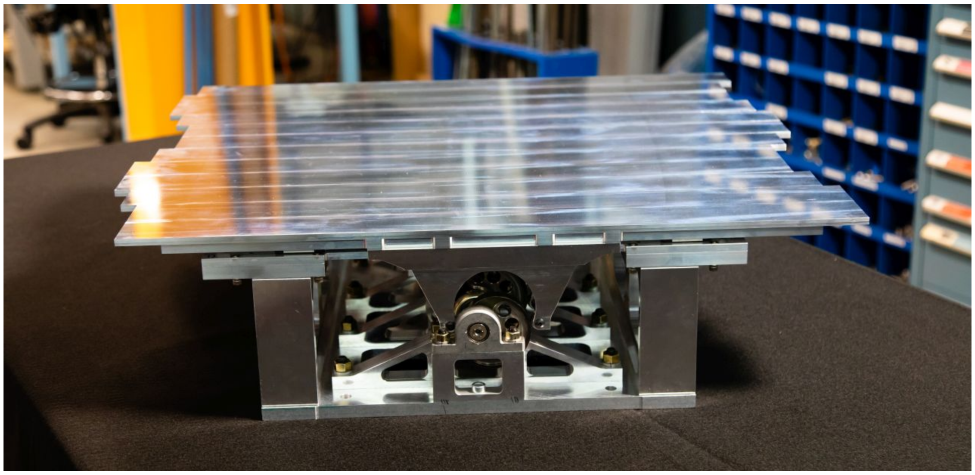

(a)

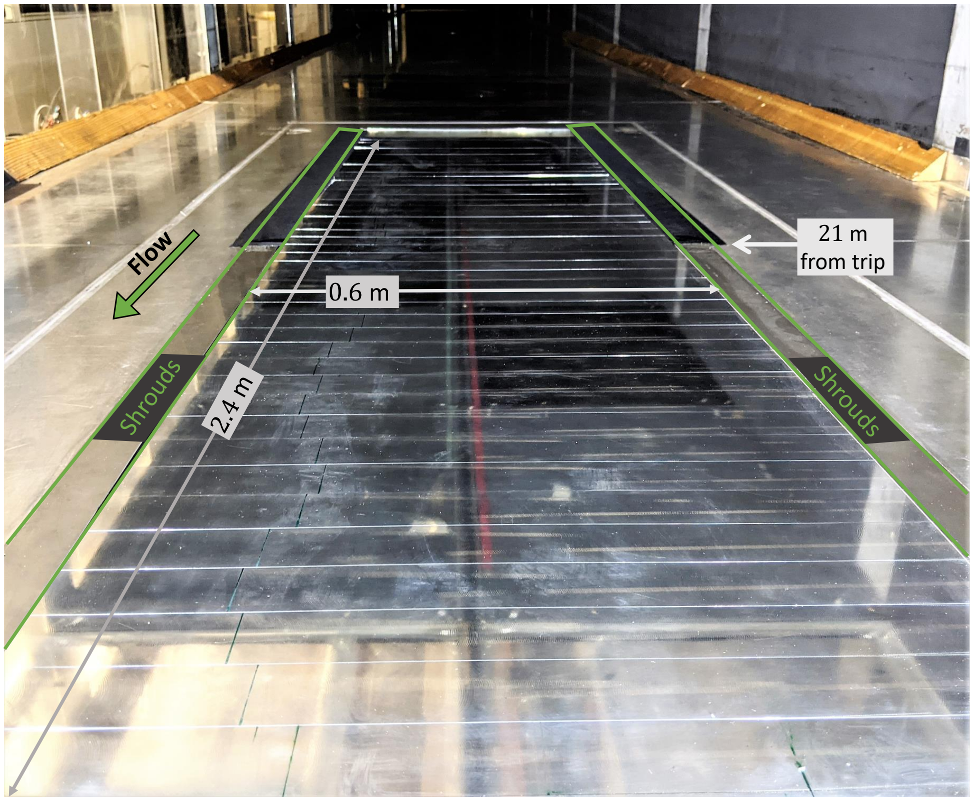

(b)

Supplementary Fig. 3. **Photograph of SATB.** (**a**) Photograph of one of the four surface-actuation test bed (SATB) machines looking down the streamwise direction. The 2λ of streamwise travelling wave generated by this machine can be seen in the slat displacement at the edge of the machine. (**b**) Photograph of SATB in the University of Melbourne wind tunnel. The four independent SATB machines are phase-synchronised to generate a 8λ (2.4 m) long streamwise travelling wave.

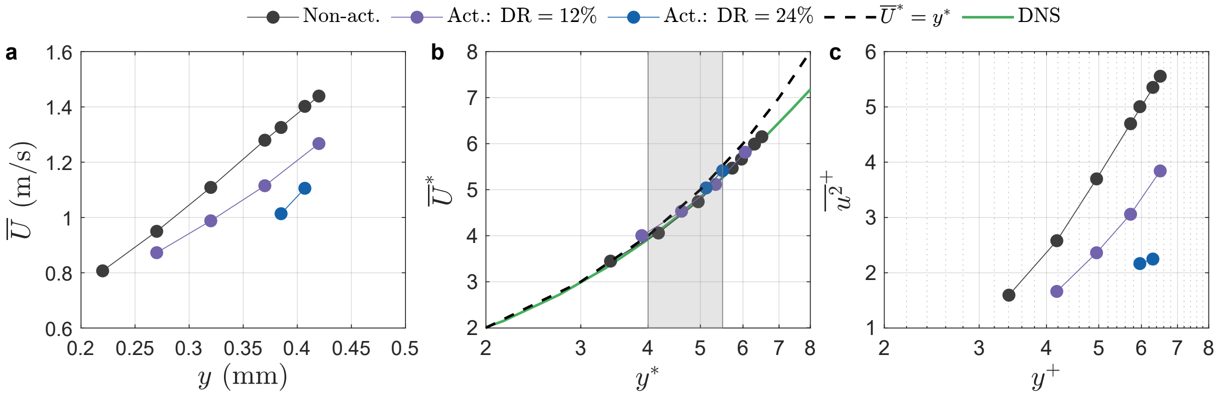

Supplementary Fig. 4. **Example hot-wire data in the viscous sublayer.** (**a**) Wall-normal profiles of mean velocity ($\overline{U}$) for non-actuated and two actuated cases. The reduction in $\overline{U}$ due to actuation is indicative of drag reduction. (**b**) Non-actuated and actuated velocity profiles from (**a**) normalized with their respective friction velocities. The grey-shaded region represents the range of wall-normal locations nominally within the linear region $\overline{U}^* = y^*$ and not contaminated by heat conduction from the sensor to the wall. Dashed line corresponds to $\overline{U}^* = y^*$ and solid green line corresponds to results from a direct numerical simulation over a smooth wall (*7*). (**c**) Variance of velocity fluctuations (turbulence intensity) normalised with the non-actuated friction velocity. In all cases, black symbols correspond to non-actuated wall and colored symbols correspond to two different actuated cases.

Experiment											LES	
Method	Sym.	Re_τ	U_∞ (m/s)	δ (m)	f (Hz)	A (m/s)	κ_x (1/m)	T^+_{osc}	A^+	κ^+_x	Sym.	Re_τ
HW, DB	●, ▲	6000	7	0.39	25	2.83	20.94	140	12	0.0014	□	951, 2003
HW	●	6000	7	0.39	10	1.13	20.94	348	4.9	0.0014	□	951, 2003
HW, DB	●, ▲	9700	11	0.39	25	2.83	20.94	362	7.8	0.0008	□	951, 2003
HW, DB	●, ▲	9700	11	0.39	15	1.7	20.94	604	4.6	0.0008	□	951, 2003
DB	▲	12800	15	0.385	25	2.83	20.94	653	5.7	0.0006		
DB	▲	12800	15	0.385	15	1.7	20.94	1100	3.5	0.0006	□	951, 2003

Supplementary Table 1. Summary of experimental parameters. Details of the flow conditions in experiments and LES with the actuation parameters adopted. The methods for the experiments include hot-wire (HW) and drag balance (DB). The symbols in the table correspond to Fig. 3 in the manuscript..

Re_τ	Actuation	$[\Delta^+_x, \Delta^+_z]$	$N_x \times N_y \times N_z$	Grid points
951	Small-eddy	[62, 31]	$144 \times 48 \times 96$	0.7 million
951	Intermediate	[30, 15]	$264 \times 96 \times 192$	4.9 million
951	Large-eddy	[30, 15]	$280 \times 96 \times 192$	5.2 million
2003	Small-eddy	[62, 31]	$224 \times 96 \times 192$	4.2 million
2003	Intermediate	[30, 15]	$470 \times 200 \times 400$	37.6 million
2003	Large-eddy	[30, 15]	$590 \times 200 \times 400$	47.2 million

Supplementary Table 2. Details of LES grid.

Supplementary References

1. Marusic, I., Mathis, R. & Hutchins, N. Predictive model for wall-bounded turbulent flow. Science **329**, 193–196 (2010).

2. Mathis, R., Marusic, I., Chernyshenko, S. I. & Hutchins, N. Estimating wall-shear-stress fluctuations given an outer region input. J. Fluid Mech. **715**, 163 (2013).

3. Chandran, D., Monty, J. P. & Marusic, I. Spectral-scaling-based extension to the attached eddy model of wall turbulence. Phys. Rev. Fluids **5**, 104606 (2020).

4. Quadrio, M. & Ricco, P. The laminar generalized Stokes layer and turbulent drag reduction. J. Fluid Mech. **667**, 135–157 (2011).

5. Gatti, D. & Quadrio, M. Performance losses of drag-reducing spanwise forcing at moderate values of the Reynolds number. Phys. Fluids **25**, 125109 (2013).

6. Jiménez, J., Hoyas, S., Simens, M. P. & Mizuno, Y. Turbulent boundary layers and channels at moderate Reynolds numbers. J. Fluid Mech. **657**, 335 (2010).

7. Sillero, J. A., Jiménez, J. & Moser, R. D. One-point statistics for turbulent wall-bounded flows at Reynolds numbers up to $\delta^+ \approx 2000$. Phys. Fluids **25** (2013).
