# Supplementary material for: An energy-efficient pathway to turbulent drag reduction
Source: Nat Commun. 2021 Oct 4;12:5805. doi: 10.1038/s41467-021-26128-8 (PMC8490469; doi:10.1038/s41467-021-26128-8)
Supplement: Supplementary file 2 — Description of Additional Supplementary Files [file 41467_2021_26128_MOESM2_ESM.docx]

Description of Additional Supplementary Files

Title: Supplementary Movie 1

Description: A movie showing the Surface Actuation Test Bed (SATB) in operation in the University of Melbourne wind tunnel facility. SATB comprising 48 slats oscillate synchronously in the spanwise direction generating a streamwise traveling wave with a total length of 2.4 m.
